# Supplementary material for: Mesoscopic theory of defect ordering–disordering transitions in thin oxide films
Source: Sci Rep. 2020 Dec 24;10:22377. doi: 10.1038/s41598-020-79482-w (PMC7759602; doi:10.1038/s41598-020-79482-w)
Supplement: Supplementary file 1 — Supplementary Information. [file 41598_2020_79482_MOESM1_ESM.pdf]

SUPPLEMENTARY MATERIALS

to

MESOSCOPIC THEORY OF DEFECT ORDERING-DISORDERING TRANSITIONS IN THIN OXIDE FILMS

Anna N. Morozovska[1], Eugene A. Eliseev[2], Dmitry V. Karpinsky[3], Maxim V. Silibin[4a,b], Rama Vasudevan[5], Sergei V. Kalinin[5 *], and Yuri A. Genenko[6†]

APPENDIX. Derivation of the Free Energy Expression

Let us denote the long-range modulation of the order parameter η, and wave vector of modulation as k_i,where i=1, 2, 3 denotes different components of vector, determining the orientations of ordering (modulation) planes, so that we suppose the following distribution η:

$$\eta = \eta_0 + \tilde{\eta}\exp[i(k_1 x_1 + k_2 x_2 + k_3 x_3)] + c.c. \tag{A.1}$$

Correspondingly, the gradient of η is

$$\frac{\partial \eta}{\partial x_j} = i k_j\, \tilde{\eta}\exp[i(k_1 x_1 + k_2 x_2 + k_3 x_3)] + c.c. \tag{A.2}$$

One could easily find from (A.1) and (A.2) the mean square average of the η gradient

$$\left\langle \frac{\partial \eta}{\partial x_i}\frac{\partial \eta}{\partial x_j}\right\rangle \cong 2|\,\tilde{\eta}|^2 k_i k_j \tag{A.3}$$

Note, that harmonic functions disappeared after the averaging.

General expression for order parameter gradient contribution the free energy density is

$$\frac{g_{ij}}{2}\frac{\partial \eta}{\partial x_i}\frac{\partial \eta}{\partial x_j} + B_{ijkl}\frac{\sigma_{ij}}{2}\left(\frac{\partial \eta}{\partial x_k}\frac{\partial \eta}{\partial x_l} + \frac{\partial \eta}{\partial x_l}\frac{\partial \eta}{\partial x_k}\right) \tag{A.4}$$

Using expressions (A.1)-(A.4) different contributions to the free energy of the system could be written as follows:

$$\left\langle g_{ij}\frac{\partial \eta}{\partial x_i}\frac{\partial \eta}{\partial x_j}\right\rangle \cong 2 g_{ij} k_i k_j |\,\tilde{\eta}|^2 \tag{A.5}$$

$$\left\langle \mathrm{B}_{ijkl}\sigma_{ij}\frac{\partial \eta}{\partial x_k}\frac{\partial \eta}{\partial x_l}\right\rangle = 2\mathrm{B}_{ijkl} k_i k_j |\,\tilde{\eta}|^2 \sigma_{ij}, \tag{A.6}$$

$$\langle \eta\rangle = \langle \eta_0 + 2|\tilde{\eta}|\cos[(\boldsymbol{kx} + \delta)]\rangle \cong \eta_0 \tag{A.7}$$

[*] Corresponding author: sergei2@ornl.gov
[†] Corresponding author: genenko@mm.tu-darmstadt.de

Taking into account (A.5)-(A.6) and using isotropic approximation, $g_{ij} = g\delta_{ij}$, the gradient and elastic contributions of free energy can be expanded on the powers of k_i. Assuming that the anisotropic Vegard tensor is diagonal (or at least can be diagonalized), that is true for many cases [1], result has the following form

$$\begin{aligned}\Delta F_{FE} = {} & gc^2(k_1^2 + k_2^2 + k_3^2)|\,\tilde{\eta}|^2 - B_{11}c^2(\sigma_{11}k_1^2 + \sigma_{22}k_2^2 + \sigma_{33}k_3^2)|\,\tilde{\eta}|^2 \\ & - B_{12}c^2(\sigma_{11}k_2^2 + \sigma_{22}k_1^2 + \sigma_{11}k_3^2 + \sigma_{33}k_1^2 + \sigma_{22}k_3^2 + \sigma_{33}k_2^2)|\,\tilde{\eta}|^2 \\ & - B_{44}c^2(\sigma_{12}k_1k_2 + \sigma_{13}k_1k_3 + \sigma_{23}k_2k_3)|\,\tilde{\eta}|^2 - \frac{s_{11}}{2}(\sigma_{11}^2 + \sigma_{22}^2 + \sigma_{33}^2) \\ & - s_{12}(\sigma_{11}\sigma_{22} + \sigma_{11}\sigma_{33} + \sigma_{22}\sigma_{33}) - \frac{s_{44}}{2}(\sigma_{12}^2 + \sigma_{13}^2 + \sigma_{23}^2) - c\left(V_{11}^c + \eta_0 V_{11}^\eta\right)\sigma_{11} \\ & - c\left(V_{22}^c + \eta_0 V_{22}^\eta\right)\sigma_{22} - c\left(V_{33}^c + \eta_0 V_{33}^\eta\right)\sigma_{33}\end{aligned} \tag{A.7}$$

Voight matrix notations is used in Eq.(A.7), and B_{ij} are the components of the fourth rank tensor in these notations, which are different from the second rank tensor, $\tilde{B}_{ij}$, introduced in the main text. Voight notations are

$$s_{1111} = s_{11}, \;\; s_{1122} = s_{12}, \;\; 4s_{1212} = s_{44}, \tag{A.8}$$

$$B_{1111} = B_{11}, \;\; B_{1122} = B_{12}, \;\; 4B_{1212} = B_{44}, \tag{A.9}$$

Modified Hooke's law could be obtained from the relation $u_{ij} = -\partial(\Delta F_{FE})/\partial\,\sigma_{ij}$:

$$u_{11} = s_{11}\sigma_{11} + s_{12}\sigma_{22} + s_{12}\sigma_{33} + \tilde{B}_{11}|\tilde{\eta}|^2c^2 + c\left(V_{11}^c + \eta_0 V_{11}^\eta\right), \tag{A.10}$$

$$u_{22} = s_{12}\sigma_{11} + s_{11}\sigma_{22} + s_{12}\sigma_{33} + \tilde{B}_{22}|\tilde{\eta}|^2c^2 + c\left(V_{22}^c + \eta_0 V_{22}^\eta\right), \tag{A.11}$$

$$u_{33} = s_{12}\sigma_{11} + s_{12}\sigma_{22} + s_{11}\sigma_{33} + \tilde{B}_{33}|\tilde{\eta}|^2c^2 + c\left(V_{33}^c + \eta_0 V_{33}^\eta\right), \tag{A.12}$$

$$u_{23} = s_{44}\sigma_{23} + \tilde{B}_{23}|\tilde{\eta}|^2c^2, \;\; u_{13} = s_{44}\sigma_{13} + \tilde{B}_{13}|\tilde{\eta}|^2c^2, u_{12} = s_{44}\sigma_{12} + \tilde{B}_{12}|\,\tilde{\eta}|^2c^2. \tag{A.13}$$

Here we used the designation for the convolution with wave vector:

$$\tilde{B}_{ij} \equiv B_{ijmn}k_mk_n$$

For instance, one has the following relations: $\tilde{B}_{11} \equiv B_{11}k_1^2 + B_{12}(k_2^2 + k_3^2)$, $\tilde{B}_{22} \equiv B_{11}k_2^2 + B_{12}(k_1^2 + k_3^2)$, $\tilde{B}_{33} \equiv B_{11}k_3^2 + B_{12}(k_1^2 + k_2^2)$, $\tilde{B}_{12} \equiv B_{44}k_1\,k_2$, $\tilde{B}_{13} \equiv B_{44}k_1\,k_3$, and $\tilde{B}_{23} \equiv B_{44}k_2\,k_3$.

The solution for the misfit of thin film with its substrate is well known. For the film with normal along X_3 one has the following relations for some of stress and strain components:

$$\sigma_{13} = \sigma_{23} = \sigma_{33} = 0 \tag{A.14}$$

$$u_{11} = u_m, \quad u_{22} = u_m, \;\; u_{12} = 0 \tag{A.15}$$

Here $u_m$is the misfit strain. For the sake of simplicity let us consider the case of only one zero component, $k_2 = 0$. Therefore, taking (A.14) and (A.15) into account, one could rewrite (A.10)-(A.13) in the following form:

$$u_m = s_{11}\sigma_{11} + s_{12}\sigma_{22} + \tilde{B}_{11}|\,\tilde{\eta}|^2 c^2 + c\left(V_{11}^c + \eta_0 V_{11}^\eta\right), \quad \text{(A.16a)}$$

$$u_m = s_{12}\sigma_{11} + s_{11}\sigma_{22} + \tilde{B}_{22}|\,\tilde{\eta}|^2 c^2 + c\left(V_{22}^c + \eta_0 V_{22}^\eta\right), \quad \text{(A.16b)}$$

$$u_{33} = s_{12}\sigma_{11} + s_{12}\sigma_{22} + \tilde{B}_{33}|\,\tilde{\eta}|^2 c^2 + c\left(V_{33}^c + \eta_0 V_{33}^\eta\right), \quad \text{(A.16c)}$$

$$u_{23} = \tilde{B}_{23}|\tilde{\eta}|^2 c^2,\; u_{13} = \tilde{B}_{13}|\,\tilde{\eta}|^2 c^2, 0 = s_{44}\sigma_{12} + \tilde{B}_{12}|\,\tilde{\eta}|^2 c^2. \quad \text{(A.16d)}$$

The solution of the system (A.16) is

$$\sigma_{11} = \frac{2u_m - (V_{11}^c + \eta_0 V_{11}^\eta)c - (V_{22}^c + \eta_0 V_{22}^\eta)c - (\tilde{B}_{11} + \tilde{B}_{22})|\,\tilde{\eta}|^2 c^2}{2(s_{11} + s_{12})} - \frac{(V_{11}^c + \eta_0 V_{11}^\eta)c - (V_{22}^c + \eta_0 V_{22}^\eta)c + (\tilde{B}_{11} - \tilde{B}_{22})|\,\tilde{\eta}|^2 c^2}{2(s_{11} - s_{12})}$$

$$\sigma_{22} = \frac{2u_m - (V_{11}^c + \eta_0 V_{11}^\eta)c - (V_{22}^c + \eta_0 V_{22}^\eta)c - (\tilde{B}_{11} + \tilde{B}_{22})|\,\tilde{\eta}|^2 c^2}{2(s_{11} + s_{12})} + \frac{(V_{11}^c + \eta_0 V_{11}^\eta)c - (V_{22}^c + \eta_0 V_{22}^\eta)c + (\tilde{B}_{11} - \tilde{B}_{22})|\,\tilde{\eta}|^2 c^2}{2(s_{11} - s_{12})}$$

$$u_{33} = \left(V_{33}^c + \eta_0 V_{33}^\eta\right)c + \tilde{B}_{33}|\,\tilde{\eta}|^2 c^2 +$$

$$+ \frac{s_{12}}{s_{11} + s_{12}}\left[2u_m - \left(V_{11}^c + \eta_0 V_{11}^\eta + V_{22}^c + \eta_0 V_{22}^\eta\right)c - \left(\tilde{B}_{11} + \tilde{B}_{22}\right)c^2|\,\tilde{\eta}|^2\right]$$

$$\sigma_{12} = -\frac{\tilde{B}_{12}|\,\tilde{\eta}|^2 c^2}{s_{44}}, u_4 = \tilde{B}_{23}|\tilde{\eta}|^2 c^2, u_5 = B_{44} k_1 k_3|\,\tilde{\eta}|^2 c^2. \quad \text{(A.17)}$$

Finally, free energy renormalization could be obtained from the Legendre transformation of the initial free energy (A.7), $\Delta\tilde{F}_{FE} = \Delta F_{FE} + \sigma_{11}u_{11} + \sigma_{22}u_{22}$, that is:

$$\Delta\tilde{F}_{FE} = gc^2(k_1^2 + k_2^2 + k_3^2)|\,\tilde{\eta}|^2 - \left(\tilde{B}_{11}\sigma_{11} + \tilde{B}_{12}\sigma_{12} + \tilde{B}_{22}\sigma_{22} + \tilde{B}_{33}\sigma_{33}\right)|\,\tilde{\eta}|^2 c^2 - c\left(\left(V_{11}^c + \eta_0 V_{11}^\eta\right)\sigma_{11} + \left(V_{22}^c + \eta_0 V_{22}^\eta\right)\sigma_{22}\right) - \frac{s_{11}}{2}(\sigma_{11}^2 + \sigma_{22}^2) - s_{12}(\sigma_{11}\sigma_{22}) - \frac{s_{44}}{2}\sigma_{12}^2 + \sigma_{11}u_m + \sigma_{22}u_m \equiv gc^2(k_1^2 + k_2^2 + k_3^2)|\,\tilde{\eta}|^2 + \frac{\left(\tilde{B}_{12}|\,\tilde{\eta}|^2 c^2\right)^2}{2s_{44}} + \frac{((V_{11}^\eta - V_{22}^\eta)\eta_0 c + (V_{11}^c - V_{22}^c)c + (\tilde{B}_{11} - \tilde{B}_{22})|\,\tilde{\eta}|^2 c^2)^2}{4(s_{11} - s_{12})} + \frac{((V_{11}^\eta + V_{22}^\eta)\eta_0 c + (V_{11}^c + V_{22}^c)c + (\tilde{B}_{11} + \tilde{B}_{22})|\tilde{\eta}|^2 c^2 - 2u_m)^2}{4(s_{11} + s_{12})} \quad \text{(A.18b)}$$

Expansion on the powers of η could be

$$\Delta\tilde{F}_{FE} = \left[g(k_1^2 + k_2^2 + k_3^2) + \frac{\{(V_{11}^\eta + V_{22}^\eta)\eta_0 c + (V_{11}^c + V_{22}^c)c - 2u_m\}(\tilde{B}_{11} + \tilde{B}_{22})}{2(s_{11} + s_{12})} + \frac{\{(V_{11}^\eta - V_{22}^\eta)\eta_0 c + (V_{11}^c - V_{22}^c)c\}(\tilde{B}_{11} - \tilde{B}_{22})}{2(s_{11} - s_{12})}\right]c^2|\tilde{\eta}|^2 + \left[\frac{(\tilde{B}_{12})^2}{2s_{44}} + \frac{(\tilde{B}_{11} - \tilde{B}_{22})^2}{4(s_{11} - s_{12})} + \frac{(\tilde{B}_{11} + \tilde{B}_{22})^2}{4(s_{11} + s_{12})}\right]c^4|\tilde{\eta}|^4 + \frac{\{(V_{11}^\eta - V_{22}^\eta)\eta_0 c + (V_{11}^c - V_{22}^c)c\}^2}{4(s_{11} - s_{12})} + \frac{\{(V_{11}^\eta + V_{22}^\eta)\eta_0 c + (V_{11}^c + V_{22}^c)c - 2u_m\}^2}{4(s_{11} + s_{12})} \quad \text{(A.19)}$$

Finally, for the case of $k_2 = 0$ and $\eta_0 = 0$ the evident form of the free energy renormalization under misfit stress appearance is

$$\Delta\tilde{F}_{FE} = \left(g - \frac{(2u_m-(V_{11}^c+V_{22}^c)c)(B_{11}+B_{12})}{2(s_{11}+s_{12})} + \frac{(V_{11}^c-V_{22}^c)(B_{11}-B_{12})}{2(s_{11}-s_{12})}c\right)c^2k_1^2|\tilde{\eta}|^2 + \left(g - \frac{(2u_m-(V_{11}^c+V_{22}^c)c)B_{12}}{s_{11}+s_{12}}\right)|\tilde{\eta}|^2c^2k_3^2 + \left(\frac{1}{4}\frac{(B_{11}+B_{12})^2c^4}{s_{11}+s_{12}} + \frac{1}{4}\frac{(B_{11}-B_{12})^2c^4}{s_{11}-s_{12}}\right)k_1^4|\tilde{\eta}|^4 + \left(\frac{(B_{12})^2c^4}{s_{11}+s_{12}}\right)k_3^4|\tilde{\eta}|^4 + \left(\frac{(B_{11}+B_{12})B_{12}c^4}{s_{11}+s_{12}}\right)k_1^2k_3^2|\tilde{\eta}|^4 + \frac{\{(V_{11}^c-V_{22}^c)c\}^2}{4(s_{11}-s_{12})} + \frac{\{(V_{11}^c+V_{22}^c)c-2u_m\}^2}{4(s_{11}+s_{12})} \qquad \text{(A.20)}$$

Note that the stabilization term proportional to fourth degree of order parameter is allowed by the symmetry and thus can be included in the free energy from the beginning. However, the material coefficient before the term is unknown and should still be defined. That is why we used the above way to re-derive the term with already defined coefficient from the entropy term. Actually, expansion on small η gives us that:

$$\frac{c\,k_BT}{2}\left[(1-\eta)\ln(1-\eta) + (1+\eta)\ln(1+\eta)\right] \approx \frac{c\,k_BT}{2}\left[\eta^2 + \frac{\eta^4}{6} + O[\eta]^6\right] \qquad \text{(A.21)}$$

The additional fourth power of η in Eq.(8a), $\gamma_{pr} = \frac{12\tilde{B}_{22}^2}{s_{11}+s_{12}}$, is related to the elimination of the elastic variables, namely elastic stresses and strains, from the initial free energy [given by e.g. above equations (A.20) and Eq.(5b)]. Mathematically, explicit dependences on the free energy coefficients and the order parameter η were obtained for stress and strains. These dependences were substituted into free energy (taking into account the Legendre transformation), which gave the renormalized energy (8b) and (9b).

---

[1] Daniel A. Freedman, D. Roundy, and T. A. Arias, Phys. Rev. B 80, 064108 (2009).
